# 

**Published:** 1871-03

**Authors:** 


### INDEX OF SUBJECTS.

ORIGINAL COMMUNICATIONS.

Fulton County Medical Society..........................................101
Bromide of Potassium in After Pains....................................108
Catheterism in the Female..............................................109
Remarks on Lucorrhœa...................................................111
Application of Pressure to Uterus in cases of Lingering Labour.........112
Treatment of Diptheria.................................................113
Treatment of Aphthæ....................................................114
Diet of Parturient Women...............................................115
Bromide of Potassium to prevent Nausea following the use of Opium.....116

EXTRACTS AND GLEANINGS.

Therapeutic Value of Chloride of Ammonium—Management of the Perineum in Labor, 117; Local use of Carbolic Acid, 118; Chloral in Asthmatic Bronchitis, 119; Subcutaneous Injection of Bubo—Treatment of Chilblains—Miasmatic Hematuria, 120; Treatment of Infantile Diarrhœa—Croton Oil in Scarlatinal Dropsy—Indian Hemp in Menorrhagia—Bilious Colic, 121; Hæmorrhoids—Nux Vomica in Dyspepsia—Nitrate of Silver for Obstinate Headache, 122; Treatment of Uterine Catarrh by Internal application of Carbolic Acid, 123; Sambucus Canadansis in Albuminuria—Nitrate of Silver in Vomiting of Pregnancy—Prescription for Opthalmia Granular Lids—Arsenic in Uterine Affections, 124; Whooping Cough treated by Choral—Treatment of Sycosis—Skin Diseases, 125; Coal Oil in Phthisis—Uses of Carbolic Acid in Syphilitic Ulcerations of the Throat—Dyspepsia, 127; Neuralgia Pill—Syphilitic Pneumonia—Creosote in Dysentery, 128; Carbolic Acid in Diptheria—Chronic Bronchitis, 129; Prescriptions for Constipation, Chronic Hepatitis, Pruritus, etc., 130; Belladonna Solution—Iodine Injections in Leucorrhœa—Remedy for Sore Nipples, 131; Strichnia in Medico-Forensic Analysis—Gargle for Ulceration of the Throat, 132.

BIOGRAPHICAL, ETHICAL, HYGENIC AND MISCELLANEOUS.

Our New Department.....................................................133
Correspondence.........................................................137
Hygenic and Miscellaneous..............................................140

EDITORIALS

Deception..............................................................147
Our Position...........................................................149
The Berks County (Pa.) Medical Society.................................151
An Interesting Letter..................................................154
The Georgia Medical Association........................................157
The American Medical Association—Our Exchanges.........................158

